# Supplementary material for: Structural insights into flagellar stator–rotor interactions
Source: eLife. 2019 Jul 17;8:e48979. doi: 10.7554/eLife.48979 (PMC6663468; doi:10.7554/eLife.48979)
Supplement: Supplementary file 1. [file elife-48979-supp1.docx]

| **Strains** | **Gene** | **Gene product** | **Motility** |
| --- | --- | --- | --- |
| Wild type | N/A | N/A | motile |
| Δ*motB* | BB0280 | stator protein | nonmotile |
| Δ*motA* | BB0281 | stator protein | nonmotile |
| *motB*-D24E | N/A | N/A | less motile |
| *motB*-D24N | N/A | N/A | nonmotile |
| *MotB^+^* | N/A | N/A | motile |
| ∆*fliL* | BB0279 | motor protein | less motile |
| CCCP | N/A | N/A | nonmotile |
