# Supplementary material for: Structural insights into flagellar stator–rotor interactions
Source: eLife. 2019 Jul 17;8:e48979. doi: 10.7554/eLife.48979 (PMC6663468; doi:10.7554/eLife.48979)
Supplement: Supplementary file 2. [file elife-48979-supp2.docx]

| **Genotype** | **Recorded camera** | **Pixel size (Å)** | **No. of subtomograms for motor alignment** | **Resolution (Å)**  **(FSC=0.5)** | **No. of subtomograms for local refinement** | **Resolution (Å)**  **(FSC=0.5)** |
| --- | --- | --- | --- | --- | --- | --- |
| WT | DDD | 2.5 | 5919 | 29 | 11380 | 20 |
| ∆*motA* | CCD | 5.7 | 889 | 47 | N/A | N/A |
| ∆*motB* | DDD | 2.5 | 3063 | 34 | 3700 | 30 |
| *MotB*^+^ | CCD | 5.7 | 911 | 43 | N/A | N/A |
| *motB*-D24E | CCD | 5.7 | 1389 | 42 | 2586 | 34 |
| *motB*-D24N | CCD | 5.7 | 1126 | 44 | 1570 | 42 |
| ∆*fliL* | CCD | 5.7 | 762 | 44 | N/A | N/A |
| CCCP | CCD | 5.7 | 791 | 45 | N/A | N/A |
